# Regional Susceptibility to TNF-α Induction of Murine Brain Inflammation via Classical IKK/NF-κB Signalling

**DOI:** 10.1371/journal.pone.0039049

**Published:** 2012-06-11

**Authors:** Adam M. H. Young, Elaine C. Campbell, Sarah Lynch, Malcolm H. Dunn, Simon J. Powis, John Suckling

**Affiliations:** 1 School of Medicine, University of St. Andrews, Fife, Scotland, United Kingdom; 2 School of Clinical Medicine, University of Cambridge, Cambridge, United Kingdom; 3 School of Physics and Astronomy, University of St. Andrews, Fife, Scotland, United Kingdom; 4 Department of Psychiatry, University of Cambridge, Cambridge, United Kingdom; Emory University, United States of America

## Abstract

It is becoming clear that inflammation plays a significant role in a number of neurological and psychiatric conditions. Post mortem brain samples in Alzheimer's disease, Parkinson's disease, amyotrophic lateral sclerosis, multiple sclerosis, schizophrenia and most recently autism spectrum condition, all exhibit neuroglial activation and inflammatory markers within the CSF. Many questions remain about the underlying molecular mechanisms. By adding the pro-inflammatory cytokine, TNF-α, to mouse brain tissue we demonstrated that the frontal lobes and temporal region, areas involved in higher functions such as memory and learning, are most susceptible to cytokine-induced inflammation via the NF-κB signalling pathway. We observed direct correlations between the volumetric increase and molecular expression indicating that therapeutic targets in these lobes may require different approaches when treating conditions with a central neuroinflammatory component.

## Introduction

Inflammation potentially plays a major role in a number of neurological and psychiatric conditions. Direct evidence for an innate inflammatory response has been demonstrated in Alzheimer's disease (AD) [1], Parkinson's disease (PD) [2], multiple sclerosis (MS) [3], amyotrophic lateral sclerosis (ALS) [3], schizophrenia [4] and most recently autism spectrum condition (ASC) [5]–[9]. Although affecting different areas, a common theme is the specificity of the inflammation, in that it is confined to certain brain regions rather than a generalised effect.

Nuclear factor kappa-light-chain-enhancer of activated B cells (NF-κB) is a family of proteins that control the transcription of DNA. It induces the expression of inflammatory cytokines and chemokines and, in turn, is induced by them [10], [11]. This establishes a positive feedback mechanism [12], which has the potential, when NF-κB becomes aberrantly active, to produce the chronic or excessive inflammation associated with several inflammatory diseases [9]. Furthermore, post mortem studies suggest that the transcription factor plays a key role in many conditions [1]–[4], [13].

Our evaluation of this process, reported here, was targeted towards the innate immune system and the activation of the classical pathway using tumour necrosis factor-α (TNF-α). It is well established that the classical NF-κB intracellular signally pathway for innate inflammatory response is based on IκB kinase (IKK) degradation by activated IKKβ (inhibitor of nuclear factor kappa-B kinase subunit beta) liberating NF-κB and allowing translocation to the nucleus where it activates the genes involved in immune responses [14].

In greater detail, the activation and nuclear translocation of NF-κB dimers (mainly p50–p65) is associated with increased transcription of genes encoding chemokines, cytokines, adhesion molecules (intercellular adhesion molecule-1, vascular cell adhesion molecule-1 and endothelial–leukocyte adhesion molecule-1), and enzymes that produce secondary inflammatory mediators and inhibitors of apoptosis [11]. The activation of such signalling pathways leads to translocation of NF-κB dimers from the cytoplasm to the nucleus [9], [10]. Proinflammatory cytokines and pathogen-associated molecular patterns, working through different receptors belonging to the tumor necrosis factor receptor and Toll-like receptor, interleukin-1 receptor superfamilies, cause activation of the IκB kinase (IKK) complex [11]. The most common form of this complex consists of the IKKα and IKKβ catalytic subunits and the IKKγ regulatory subunit. Like NF-κB proteins IKKα and IKKβ undergo homo- and hetero-dimerization. In the classical NF-κB signalling pathway, the activated IKK complex, predominantly acting through IKKβ in an IKKγ-dependent manner, catalyzes the phosphorylation of IκBs (at sites equivalent to Ser32 and Ser36 of IκBα), polyubiquitination (at sites equivalent to Lys21 and Lys22 of IkBα) and subsequent degradation by the 26S proteasome [15]. The released NF-κB dimers (in this pathway, most commonly the p50–p65 dimer) translocate to the nucleus, bind DNA and activate gene transcription [15]. Deficiencies in NF-kB p65 and IKKβ result in a marked increase in susceptibility of the organism to infections, once the embryonic lethality associated with these deficiencies is prevented [16]–[17].

In this current study we have addressed the question as to whether three distinctly different regions of the murine brain that are known to be affected by inflammatory brain disease to different extents respond to cytokine induced inflammation, and whether this correlates with NF-κB expression. In doing so we applied a novel pressure chamber system to assess the differential inflammatory response of cortical lobes based on volume as well as traditional histological analysis.

## Materials and Methods

This study was carried out in strict accordance with United Kingdom Home Office guidelines on the Care and Use of Laboratory Animals. The protocol was approved by the University Teaching and Research Ethics Committee (UTREC) of the University of St Andrews (Reference Number: MD4637). No experimental procedures were performed on live animals. All animals were sacrificed by schedule 1 cervical dislocation by trained personnel, and all efforts were made to minimize suffering.

Primary neurons were stimulated with TNF-α to measure NF-κB expression using immunofluorescence and immunoblotting techniques in order to gain an impression of the molecular response *in vitro*. This response was then measured *in vivo* to observe the contrast in NF-κB expression between the two preparations and as such the novelty of our model in assessing inflammation in whole tissue. Upon recognition that the NF-κB response was significantly different in the three lobes measured we then measured the volumetric change in these areas and assessed the potential association with the transcription factor.

### Primary neuron culture and stimulation with TNF-α

Pregnant time-mated CD1 mice were sacrificed by schedule 1 cervical dislocation after 14 days gestation and the cortices dissected from the embryos, under sterile conditions as described by Oldreive et al., [18]. The tissue was trypsinised and a single cell suspension plated on 0.001% (w/v) polylysinecoated 6-well plates (Nunc) in MEM supplemented with 10% horse serum and 10% fetal calf serum (FCS). After 24 hours the medium was switched to MEM supplemented with serum replacement 2 (Sigma, UK) and 18 μM 5-fluorodeoxyuridine to inhibit the proliferation of non-neuronal cells. Primary cortical neuron cultures (>99% pure) were incubated for a further 18 hours and stimulated with 10 ng/ml TNF-α for 2 hours before antigen positive cells were counted in each sample to quantify the intensity of anti-p65 signal in the nucleus, providing a measurement of nuclear translocation of NF-κB p65 and thus the active state of the molecule.

### Western Immunodetection of primary neurons

Protein samples run on SDS-polyacrylamide gels were electroblotted to nitrocellulose membranes (Schleicher & Schuell, Germany), blocked with 5% non-fat dry milk in PBS with 0.1% Tween 20 (PBST) and probed with a 1∶1000 dilution of anti-p65 antibody (Santa Cruz Biotechnology). PBST washed membranes were then incubated with HRP-conjugated goat anti-rabbit antisera (Sigma, UK), and developed with enhanced chemiluminescence reagents (Pierce, UK). Signal was detected using a LAS 3000 image analyzer (Fujifilm, Japan) and bands quantified using ImageJ software.

### Isolation of tissue cultures

Adult CD1 mice (33–40 g) were sacrificed by schedule 1 cervical dislocation as above. Brains were extracted and dissected according to Hagihara et al., [19] to provide cerebellum, hippocampus with associated cortex (temporal region) and frontal lobe preparations. These were allowed to recover for 3 hours in 5% heat inactivated foetal calf serum (FCS) in RPMI 1640 medium (Gibco, UK) at 37°C, and thereafter stimulated with 10 ng/ml TNF-α (R&D Systems, UK) [20]. For individual cell preparations 3 mm^3^ of tissue was mechanically disrupted and trypsinised (Sigma, UK) for 10 minutes to provide a single cell suspension. RPMI 1640 medium with 5% FCS was added to inhibit trypsin activity. Isolated cells were stimulated with 10 ng/ml TNF-α for up to 4 hours and 96 hours in separate preparations. For inhibition studies, 1 μg/ml IKK Inhibitor (Santa Cruz Biotechnology, Inc., Santa Cruz, CA) or 150 μg/ml sodium salicylate (Santa Cruz Biotechnology) were added.

### Dissociation of cytosolic and nuclear material

Tissue samples were processed for nuclear and cytosolic extraction using two separation buffers. The tissue was homogenized in 10 volumes of buffer 1 (Tris 10 mM, NaH2PO4 20 mM, EDTA 1 mM, pH 7.8 PMSF 0.1 mM, pepstatin 10 μg/ml, and leupeptin 10 μg/ml). Homogenate was incubated for 20 min and osmolarity restored by adding 1/20 volume of KCl 2.4 M, 1/40 volume of NaCl 1.2 M, 1/5 volume sucrose 1.25 M. Samples were spun for 5 min at 3,500 rpm, supernatant removed and pellet resuspended on 0.6 M sucrose and spun for a further 10 min at 10,000 rpm.

Subsequently, the supernatant was diluted in buffer 2 (imidazole 30 mM, KCl 120 mM, NaCl 30 mM, NaH2PO4, sucrose 250 mM pH 6.8, protease inhibitors pepstatin 10 μg/ml and leupeptin 10 μg/ml) and spun again at 3,500 rpm for 15 min. The resultant pellets contained the remaining nuclear proteins, and the supernatants the cytosolic proteins.

### Immunofluorescence microscopy and Western Immunodetection of tissue cultures

A 3 mm^3^ volume of tissue was mechanically dissociated and fixed in 4% formaldehyde in PBS. Cells were stained with anti-Beta III Tubulin (1∶10,000), a microtubule element antibody targeted exclusively in neurons, and anti-p65 antibody (1∶50 dilution) in buffer containing 0.2% saponin for 60 minutes. Samples were then washed and incubated with FITC & Texas Red labelled second stage. This was followed by a final 5 minute wash and slides were mounted with 4′,6-diamidino-2-phenylindole (DAPI) with Vectashield (Vector Labs Ltd, UK) to identify cell nuclei.

One hundred neurons from each sample were selected from 10 visual fields covering the full area of the tissue slice within each of which 10 neurons were selected at random, blinded from group (i.e., control or TNF-α stimulated). Antigen positive cells were counted in each sample using the rating scale of Schmidt and Bankole [21], to quantify the intensity of anti-p65 signal in the nucleus providing a measurement of nuclear translocation of NF-κB p65 and thus the active state of the molecule. Each cell was given a rating of fluorescence from 0 (negative) to 3+ (most intense). A fluorescence scale of 3+ was calibrated on the intense green fluorescence exhibited by NF-κB antigen in HeLa cell controls. Scoring was verified by image J analysis.

Western Immunodetection was performed as described previously.

### Volume measurements and Pressure chamber

Brain volumes were measured using a vertically-mounted travelling microscope to measure the height of liquid in a 15 ml test tube displaced by the addition of brain tissue.

For Pressure Chamber experiments tissue was added to a single well plate containing RPMI 1640 medium with 5% FCS at 37°C with or without 10 ng/ml TNF-α for up to 96 hours. Wells were sealed with parafilm and pierced with a capillary tube. The volume of fluid in the capillary was calculated from the height rise and the diameter of the capillary tube. Measurements were normalised as a percentage of the initial volume. Inflammation of the tissue was confirmed by hematoxylin and eosin (H&E) staining and observation of cell morphology. Full details are given in Table 1.

**Table 1:** Procedural details of volumetric measurements.

Step 1: Dissection of mouse brain in ventilated hood
Step 2: Fill a standard test tube with 15ml of RPMI 1640 medium with 5% FCS at 37°C
Step 3: Calibrate vertically-mounted travelling microscope to the meniscus
Step 4: Place brain tissue into test tube to displace fluid
Step 5: Measure the displaced volume by calibrating the travelling microscope to the new height of the meniscus
Step 6: Remove the brain tissue and place in a single well plate containing RPMI 1640 medium with 5% FCS at 37°C at leave to rest in the incubator for 1 hour.
Step 7: Add solution to dish and cover with sterile parafilm.
Step 8: Peirce parafilm with a pre-autoclaved capillary tube.
Step 9: Fluid will displace up the tube, allow to settle and mark the outside of the capillary.
Step 10: Place in incubator for a set time and mark the new fluid height. This can be continued repeatedly over multiple time points.
Step 11: Knowledge of the length fluid has travelled over a set time will allow calculation of displaced volume. This can now be presented as a percentage increase of initial mass.

### Statistics

Statistical testing was performed with Analysis of Variance and Tukey-Kramer procedure for multiple comparisons using Sigma Plot 5. Data was considered significant at p<0.05. Data was checked for normal distribution using the K-sample Anderson–Darling test. Unless indicated, all data are expressed as mean ± standard error across the samples.

## Results

### TNF-α induces p65 translocation in primary neuron cultures

**Figure 1:**
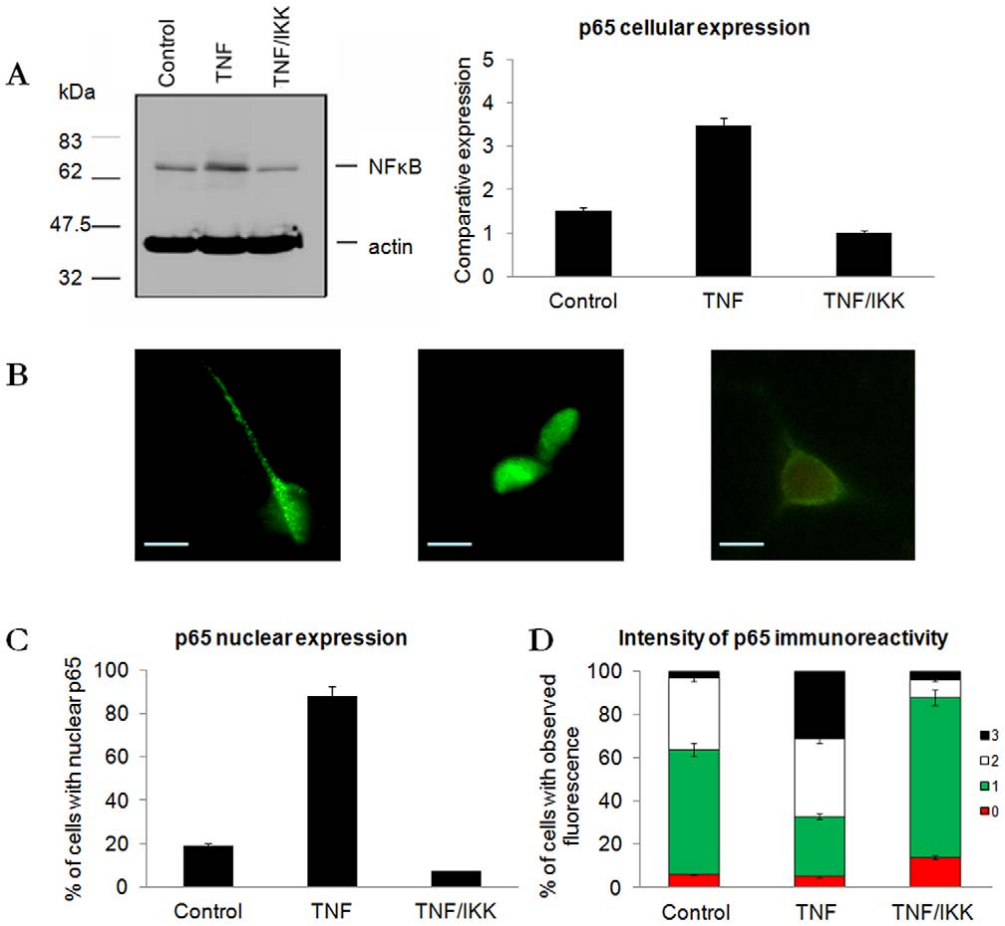
Immunofluorescent staining of primary cortical neurons. A: p65 expression in primary cortical neurons, showing increased expression with TNF-α and decreased expression with IKK Inhibitor (F = 177.79; p<0.001; df = 2,12). B: NF-κ B expression in a control neuron, using anti-p65 antibody and FITC labelled secondary, NF-κ B expression in a TNF-α stimulated neuron and NF-κ B expression in a TNF-α stimulated neuron with NF-κ B inhibitor. C: The percentage of neurons with anti-p65 staining in the nucleus (F = 30.63; p<0.001; df = 2,14), and D: the levels of fluorescence in each sample where fluorescence is rated between 0 and 3 on the scale bar (F = 17.65; p<0.002; df = 2,57). Molecular weight markers are in kilodaltons (kDa).

Primary cortical neuronal cultures were studied to calibrate the response to stimulation with 10 ng/ml TNF-α and inhibition with 1 μg/ml IKK. Whole cell NF-κB expression analysis was performed by immunoblotting with primary neurons stimulated with TNF-α demonstrating a 2-fold increase in NF-κB expression (figure 1A; F = 177.79; p<0.001; df = 2,12). After stimulation with TNF-α there was increased NF-κB p65 nuclear localisation when compared to untreated cultures (figure 1B–C; F = 30.63; p<0.001; df = 2,12, figure 1D; F = 17.65; p<0.002; df = 2,57).

IKK inhibitor prevented TNF-α mediated p65 nuclear translocation when cultures were exposed to both simultaneously. Due to the strong inhibitory properties of IKK inhibitor we also examined its potential to reverse inflammation. TNF-α stimulated primary neurons were washed and further stimulated with TNF-α with or without IKK Inhibitor. Although NF-κB effectively translocated to the nucleus in the first 2 hour period, after the wash out IKK inhibitor returned nuclear NF-κB values back to that previously observed (data not shown).

### TNF-α induces differential NF-κB expression and nuclear localisation in different brain regions

Having established nuclear translocation of NF-kB in neuronal clusters we next examined, by immunoblotting, whether levels of the NF-κB p65 following TNF-α stimulation of whole brain tissue sections were dependent on location.

**Figure 2:**
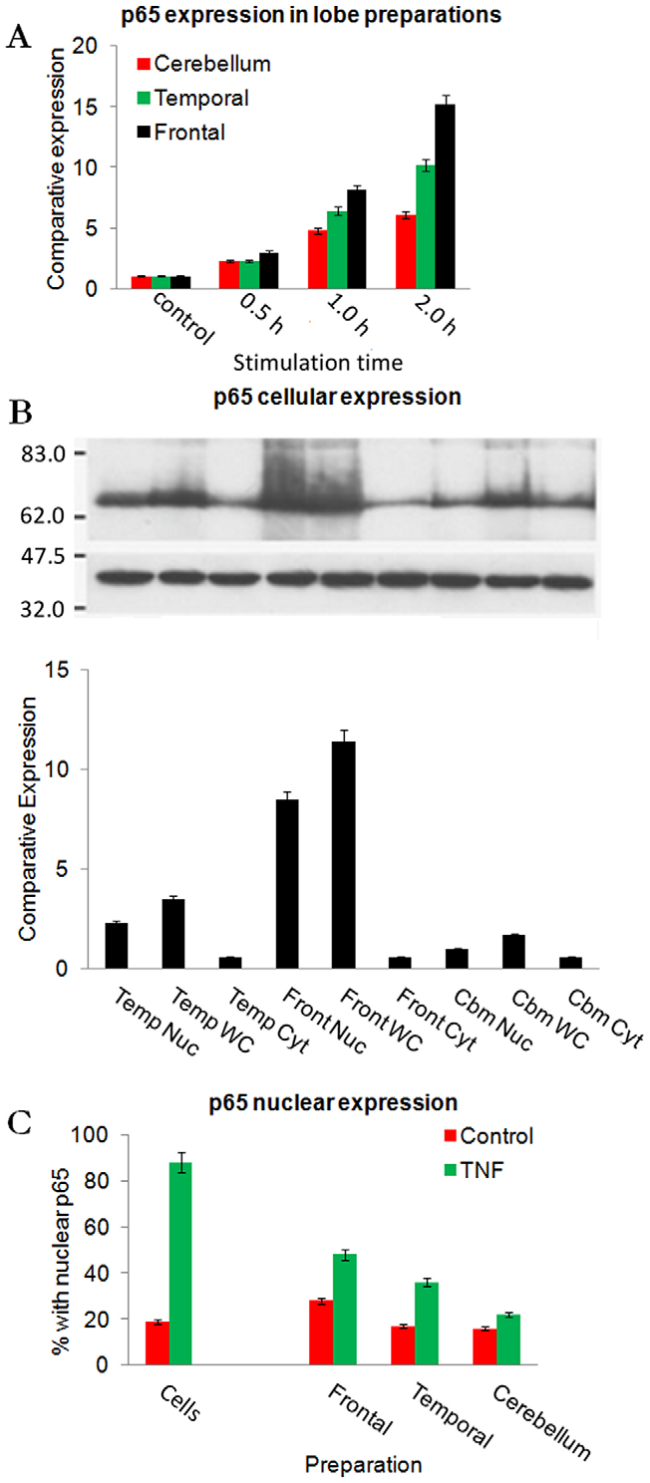
Western Blot analysis of p65 subunit of NF-κB. A: NF-κB p65 expression after stimulation with TNF-α for 0, 0.5, 1 and 2 hours. Frontal lobe (Front), Temporal region (Temp) and Cerebellum were examined (F = 8.57; p = 0.012; df = 2,57). B: Western Blot analysis of p65 subunit of NF-κB nuclear vs. cytoplasmic analysis after 1 hour of TNF-α stimulation, comparing whole cell expression (WC) to nucleus (Nuc) and cytoplasmic (Cyt) preparations, data is significant after 1 hour of stimulation (F = 5.56; p = 0.043; df = 2,27). Calibration bars are 10 µm. C: The percentage of neurons with anti-p65 staining in the nucleus, comparing *in vivo* frontal lobe, temporal region and cerebellum neurons with the aggregate of all cells (F = 20.99; p<0.001; df = 3,36). Molecular weight markers are in kilodaltons (kDa).

NF-κB p65 was upregulated with time, (data significant after 1/2 hour, figure 2A; F = 8.57; p = 0.012; df = 2,57). Interestingly, the frontal lobes and temporal region of the brain demonstrated up-regulated NF-κB p65 to a greater extent in comparison to the cerebellum. The frontal lobe and temporal region sections expressed 15-fold and 10-fold more NF-kB p65 respectively, whereas cerebellum tissue demonstrated only a 6-fold increase.

To confirm the subcellular localization of p65 in our samples we immunoblotted whole cell, cytoplasmic, and nuclear extracts. Localization of the transcription factor was mainly within the nucleus 1 hour after TNF-α stimulation (figure 2B; F = 5.56; p = 0.043; df = 2,27). We also analysed the translocation of NF-κB p65 into the nucleus using immunofluorescence of mechanically dissociated neural tissue using a rating scale based on Schmidt and Bankole [21] grading p65 fluorescence in the nucleus from 0 to 3, with the scoring system also being verified by image J analysis.

After 2 hours of stimulation from the one hundred randomly sampled neurons from each sample, nuclear localization of NF-κB p65 occurred in 48±4% of frontal lobe neurons compared to 28±3% in controls, 36±3% of temporal region neurons compared to 17±2% of controls, and 22±3% in cerebellum tissue compared to 16±2% in controls. These results were significantly lower than in the primary neuron cultures where TNF-α stimulation induced nuclear localisation in 88±7% of cells compared to 19±4% in controls (figure 2C; F = 20.99; p<0.001; df = 2,36).

### NF-κB expression and localisation correlates with brain tissue inflammation

Using the pressure chamber system and histological investigation we then sought direct evidence that the spatially varying distribution of NF-κB expression was related to inflammation.

**Figure 3:**
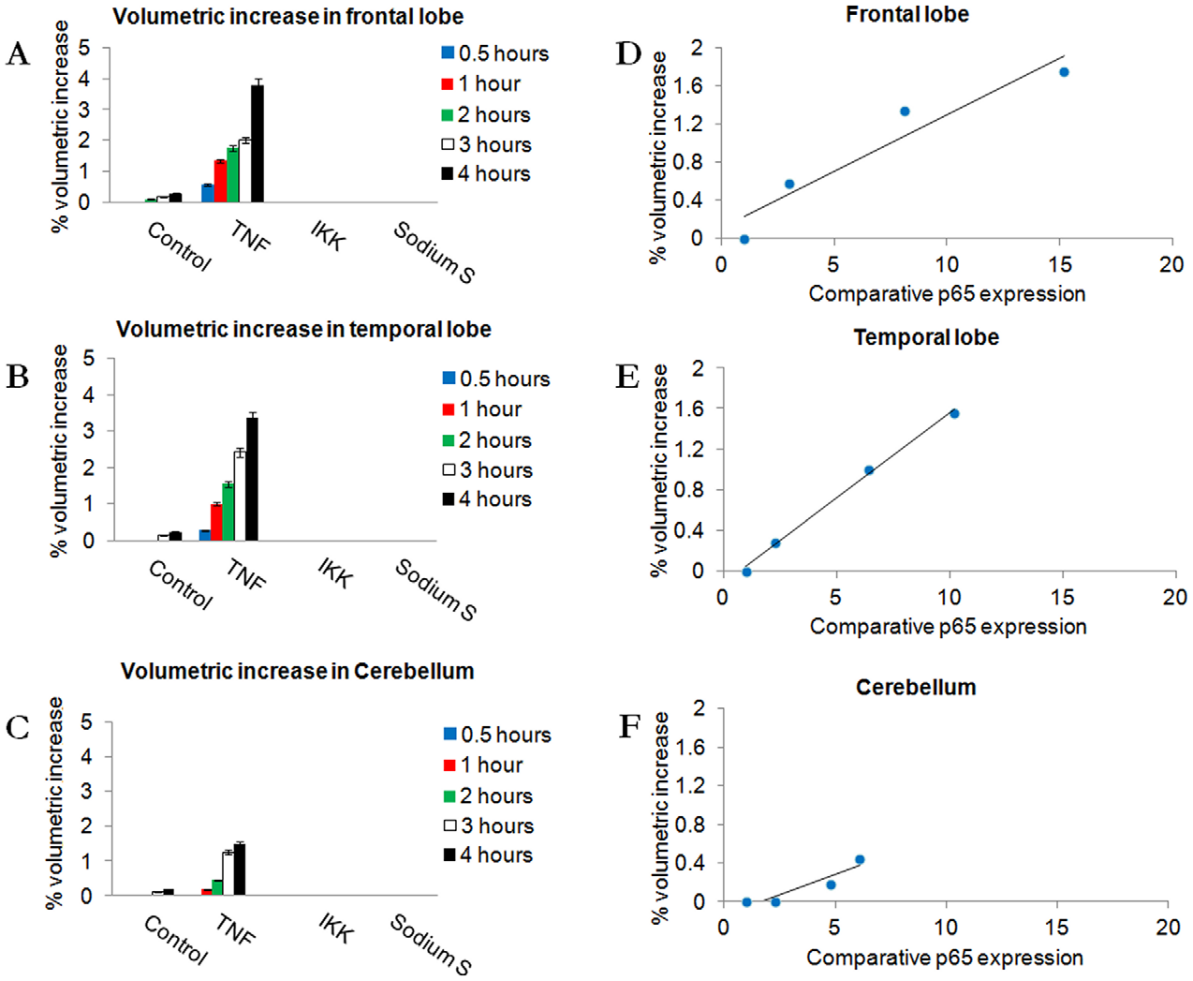
Quantification of inflammation induced by TNF-α. Tissue inflammation in Control (Ctrl), 10 ng/ml TNF-α (TNF), TNF-α with IKK Inhibitor (IKK), and TNF-α with sodium salicylate samples (Sodium S). A–C: Measurements at 0.5, 1 and 2 hours stimulation and after TNF-α removal (3 and 4 hours). Results are presented as for figure 1, data is significant after 2 hours of stimulation (F = 22.53; p = 0.001; df = 3,96). D-F: Plot of % volumetric increase of tissue vs. normalized overall expression of NF-κB p65 from quantification of Western blot samples with a superimposed line of linear regression.

Tissue was stimulated with 10 ng/ml TNF-α for 2 hours and then washed to remove TNF-α. Volumetric changes were measured at 0.5, 1 and 2 hours post-stimulation and at 1 and 2 hours post-washing. Volume change continued to occur steadily after the removal of the cytokine (figure 3A–C; F = 22.53; p = 0.001; df = 3,96), indicating an initial short stimulus triggers the inflammatory response which continues after the initial stimulus is withdrawn. Volume increases demonstrated a strong correlation with NF-kB response, the frontal lobe (figure 3D; r^2^ = 0.9126; F = 65.25; p = 0.003; df = 3,96) and temporal region (figure 3E; r^2^ = 0.9964; F = 34.96; p = 0.007; df = 3,96) of the brain demonstrated the greatest increase relative to the cerebellum (figure 3F; r^2^ = 0.8608; F = 118.89; p = 0.001; df = 3,96).

**Figure 4:**
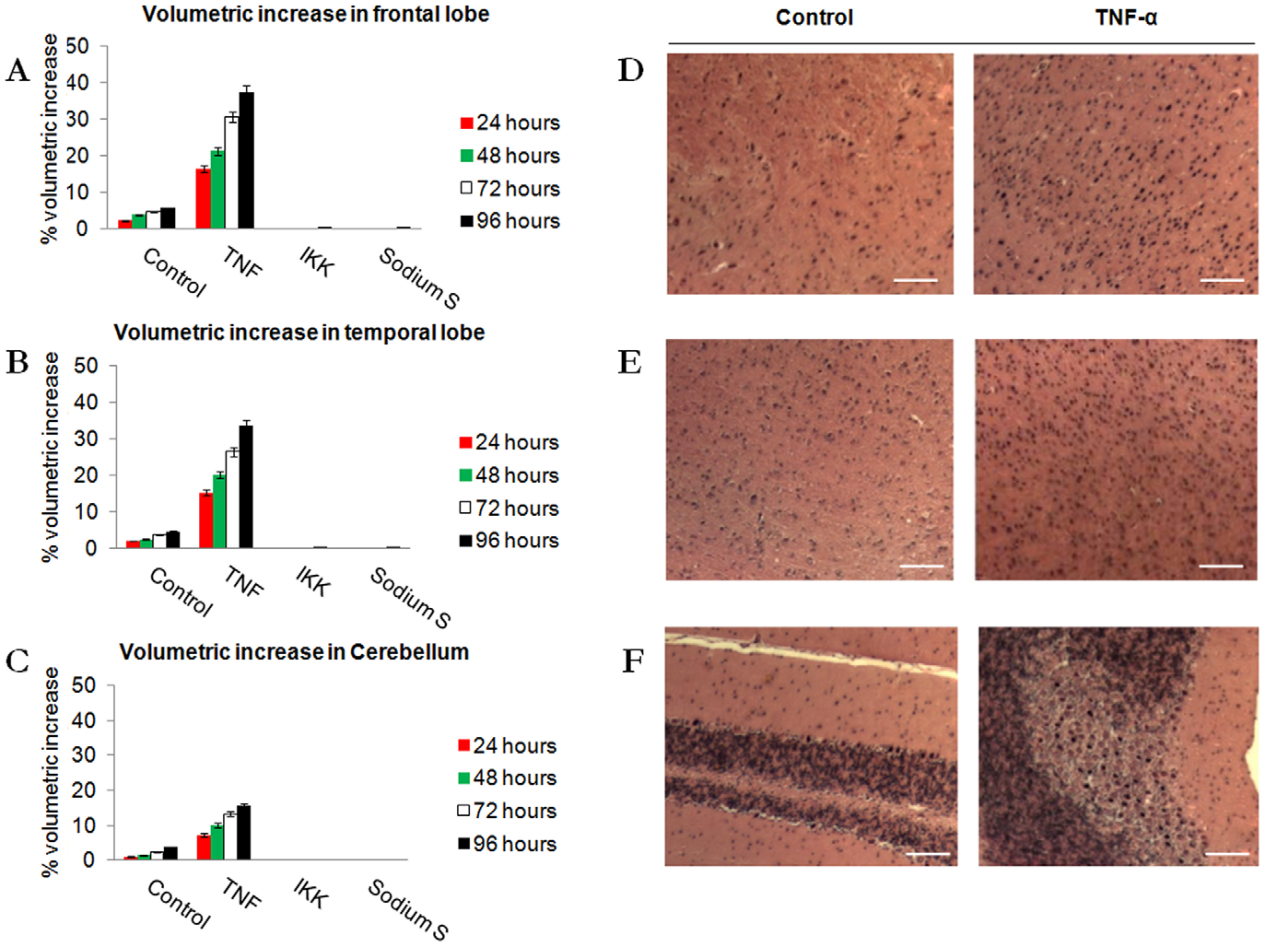
Quantification of inflammation induced by TNF-α. Tissue inflammation in Control (Ctrl), 10 ng/ml TNF-α (TNF), TNF-α with IKK Inhibitor (IKK), and TNF-α with sodium salicylate samples (Sodium S). A–C: Measurements at 24, 48, 72 and 96 hour periods in A: frontal lobe, B: temporal region, and C: cerebellum. Data is significant after 24 hours of stimulation. Results are presented as a percentage increase of original value (F = 816.22; p<0.001; df = 3,96). D–F: Confirmation of inflammation with hematoxylin and eosin (H&E) stain. Tissue stimulated with TNF-α 10 ng/ml in frontal lobe (D), temporal region (E) and cerebellum (F). Calibration bars are 50 µm.

Over a 96 hours, stimulation with TNF-α initiated an inflammatory response that produced a volume increase in excess of 30% in the whole brain preparations (not shown), with greatest changes in the sub-dissected frontal lobes and temporal region preparations (figure 4A–C; F = 816.22; p<0.001; df = 3,99). These changes corresponded with morphological changes within the tissue itself. The tissue stimulated TNF-α demonstrating a higher cellular content, representative of inflammation (figure 4D–F). This suggests that the frontal lobes and temporal region of the murine brain appear more susceptible to neuroinflammation in comparison to the other brain regions studied here.

To determine if NF-κB played a role in this inflammation, tissues were also stimulated with TNF-α in the presence of 1 μg/ml IKK Inhibitor or 150 μg/ml sodium salicylate [20]. Both IKK Inhibitor and sodium salicylate inhibited the inflammation of brain tissue in short and long term cultures, implicating IKK/NF-κB signaling in the inflammation (figure 3&4A–C).

## Discussion

NF-κB is found in almost all cells types and plays a significant role in a major inflammatory pathway. These data confirm that NF-κB is strongly expressed and translocates to the nucleus of neurons in culture. A key result in this experiment is that the level of expression and nuclear translocation of NF-κB is dependent on the location of neurons in the neocortex or cerebellum. This is linked to an inflammatory response by volume increases, histological analysis and, finally, to correlations with NF-κB expression. This microscopic-to-macroscopic succession of evidence provokes the, to our knowledge, novel conclusion that three distinctly different areas of the murine brain respond to inflammatory stimulus to varying degrees.

TNF-α was specifically selected to activate the classical NF-κB signalling cascade of the innate immune system [14] and to demonstrate nuclear translocation of NF-κB in primary cortical neurons. Our main focus was to examine the holistic response to inflammation in the brain areas described and to justify our model by comparing the level of molecular expression between in tissue model and cellular assays. In doing so we observed a significantly lower level of nuclear translocation in tissue throughout all areas of the brain demonstrating the importance of analysing NF-κB expression in cells in tissue assay.

Assessment of NF-κB p65 expression was performed in frontal cortex, temporal region and cerebellum. Frontal lobe and temporal region sections appeared to be the most affected (15-fold and 10-fold more NF-κB p65 respectively) whereas cerebellar tissue expressed a 6-fold increase. Although it has been previously recognised by Glass et al [3] that NF-κB expression is upregulated in the substantia nigra, basal forebrain, brain stem and spinal cord playing a role in inflammation in PD, AD and ALS respectively, this experiment shows that it can also be activated to greater and lesser extents within other neural structures concurrently by inducing an inflammatory process. In addition, not only is NF-κB present in aberrant quantities, nuclear translocation of the p65 subunit suggests that NF-κB is modulating gene transcription.

The explanation for the differential inflammation of brain regions is most likely to be found in the associations between microglia density and receptor expression. Whereas microglia are known to occupy all brain areas, differences in their cellular density between different brain regions under physiological conditions can be as high as five-fold in mouse [22]. Microglia are most numerous in the telencephalon, followed by diencephalon, mesencephalon and rhombencephalon containing the lowest density of microglia. Nevertheless, where we have observed an inflammatory susceptibility gradient of frontal lobe > temporal region > cerebellum, Lawson et al. [22] found that in fact the hippocampus contained more microglia that the frontal lobe. From this we deduce that the response we have witnessed is a result of receptor expression and cell signalling cascades. The density of microglia at rest has been confirmed to be higher in the temporal region [23]. However, stimulation of these areas with lipopolysaccharide (LPS) demonstrated a greater response in the frontal lobe in keeping with our observations in this experiment. Whilst possible mechanisms for this effect were not explored, it is well documented that LPS activates the nuclear translocation of NF-κB via Toll-like-receptor 4 (TLR4) [24].

A key result is the correlation between NF-κB expression and tissue volume increase. We observed that the frontal lobe and temporal region demonstrated the greatest inflammatory response following TNF-α stimulation. The frontal cortex is primarily associated with developing personality and regulating appropriate behaviours by providing an inhibitory function [25] and can be particularly affected in individuals with neuroinflammatory conditions [26]–[29]. In particular NF-κB was observed to be increased 2.9-fold higher in post-mortem samples from donors with ASC compared to matched controls [13]. Generally neuroinflammatory responses have been linked to cognitive decline [30], memory loss [31] and impairment of co-ordination [32], as well as psychiatric manifestations [12], [33] via the disruption of neural signalling. However the molecular mechanism for this inflammation has yet to be defined. We suggest that the induction of inflammation via the NF-κB signalling cascade is potentially responsible for localised neuroinflammation in higher areas of the brain leading to behavioural and clinical symptoms in a range of disorders.

In conclusion, we have reported a novel *in vitro* model for quantifying brain tissue inflammation which allows the study of inflammatory quantification and correlation with the molecular pathways involved. We have thus been able to demonstrate the regional variation of inflammation in the brain and a potential role for NF-κB. These observations suggest that NF-κB could contribute a significant role in the inflammatory pathogenesis of regional disease and raises the question of whether further consideration of the location of inflammation within the brain could be used to fine tune anti-inflammatory therapy.

## References

[1] Akiyama H (1994). Inflammatory response in Alzheimer's disease.. Tohoku J Exp Med.

[2] Block ML, Hong JS (2005). Microglia and inflammation-mediated neurodegeneration: Multiple triggers with a common mechanism.. Prog Neurobiol.

[3] Glass CK, Saijo K, Winner B, Marchetto MC, Gage FH (2010). Mechanisms underlying inflammation in neurodegeneration.. Cell.

[4] Meyer U, Feldon J, Dammann O (2011). Schizophrenia and autism: both shared and disorder-specific pathogenesis via perinatal inflammation?. Pediatr Res.

[5] Pardo CA, Vargas DL, Zimmerman AW (2005). Immunity, neuroglia and neuroinflammation in autism.. Int Rev Psychiatry.

[6] Vargas DL, Nascimbene C, Krishnan C, Zimmerman AW, Pardo, C A (2005). Neuroglial activation and neuroinflammation in the brain of patients with autism.. Ann Neurol.

[7] Zimmerman AW, Jyonouchi H, Comi AM, Connors SL, Milstien S, Varsou A, Heyes MP (2005). Cerebrospinal fluid and serum markers of inflammation in autism.. Pediatr Neurol.

[8] Herbert MR (2008). Learning from the autism catastrophe: key leverage points.. Altern Ther Health Med.

[9] Barnes PJ, Karin M (1997). Nuclear factor-kappaB: a pivotal transcription factor in chronic inflammatory diseases.. N Engl J Med.

[10] Pahl HL (1999). Activators and target genes of Rel/NF-kappaB transcription factors.. Oncogene.

[11] Perkins ND (2004). NF-kappaB: tumor promoter or suppressor?. Trends Cell Biol.

[12] Hayden MS, Ghosh S (2004). Signaling to NF-kappaB.. Genes Dev.

[13] Young AM, Campbell E, Lynch S, Suckling J, Powis J (2011). Aberrant NF-kappaB expression in autism spectrum condition: a mechanism for neuroinflammation.. Front Psychiatry.

[14] Bonizzi G, Karin M (2004). The two NF-kappaB activation pathways and their role in innate and adaptive immunity.. Trends Immunol.

[15] Ghosh S, Karin M (2002). Missing pieces in the NF-kappaB puzzle.. Cell.

[16] Alcamo E, Mizgerd JP, Horwitz BH, Bronson R, Beg AA, Scott M, Doerschuk CM, Hynes RO, Baltimore D (2001). Targeted mutation of TNF receptor I rescues the RelA-deficient mouse and reveals a critical role for NF-kappa B in leukocyte recruitment.. J Immunol.

[17] Senftleben U, Cao Y, Xiao G, Greten FR, Krähn G, Bonizzi G, Chen Y, Hu Y, Fong A, Sun SC, Karin M (2001). Activation by IKKalpha of a second, evolutionary conserved, NF-kappa B signaling pathway.. Science.

[18] Oldreive CE, Doherty GH (2010). Effects of tumour necrosis factor-alpha on developing cerebellar granule and Purkinje neurons in vitro.. J Mol Neurosci.

[19] Hagihara H, Toyama1 K, Yamasaki N, Miyakawa T (2009). Dissection of Hippocampal Dentate Gyrus from Adult Mouse.. J Vis Exp.

[20] Anderson SW, Bechara A, Damasio H, Tranel D, Damasio AR (1999). Impairment of social and moral behavior related to early damage in human prefrontal cortex.. Nat Neurosci.

[21] Schmidt EL, Bankole RO (1965). Specificity of immunofluorescent staining for study of Aspergillus flavus in soil.. Appl Microbiol.

[22] Lawson LJ, Perry VH, Dri P, Gordon S (1990). Heterogeneity in the distribution and morphology of microglia in the normal adult mouse brain.. Neuroscience.

[23] Kim WG, Mohney RP, Wilson B, Jeohn GH, Liu B, Hong JS (2010). Regional Difference in Susceptibility to Lipopolysaccharide-Induced Neurotoxicity in the Rat Brain: Role of Microglia.. J Neurosci.

[24] da Silveira Cruz-Machado S, Carvalho-Sousa CE, Tamura EK, Pinato L, Cecon E, Fernandes PA, de Avellar MC, Ferreira ZS, Markus RP (2010). TLR4 and CD14 receptors expressed in rat pineal gland trigger NFKB pathway.. J Pineal Res.

[25] Wing L, Gould J (1979). Severe impairments of social interaction and associated abnormalities in children: epidemiology and classification.. J Autism Dev Disord.

[26] Viel TA, Buck HS (2011). Kallikrein-kinin system mediated inflammation in Alzheimer's disease in vivo.. Curr Alzheimer Res.

[27] Terada T, Obi T, Yoshizumi M, Murai T, Miyajima H, Mizoguchi K (2011). Frontal lobe-mediated behavioral changes in amyotrophic lateral sclerosis: Are they independent of physical disabilities?. J Neurol Sci.

[28] Hollander E, Wang AT, Braun A, Marsh L (2009). Neurological considerations: autism and Parkinson's disease.. Psychiatry Res.

[29] Widera D, Mikenberg I, Kaus A, Kaltschmidt C, Kaltschmidt B (2006). Nuclear Factor-kappaB controls the reaggregation of 3D neurosphere cultures in vitro.. Eur Cell Mater.

[30] Zilbovicius M, Boddaert N, Belin P, Poline JB, Remy P, Mangin JF, Thivard L, Barthélémy C, Samson Y (2000). Temporal lobe dysfunction in childhood autism: a PET study.. Am J Psychiatry.

[31] Viel TA, Buck HS (2011). Kallikrein-kinin system mediated inflammation in Alzheimer's disease in vivo. Curr Alzheimer Res..

[32] Zindler E, Zipp F (2010). Neuronal injury in chronic CNS inflammation. Best Pract Res Clin Anaesthesiol..

[33] Konradi C, Sillivan SE, Clay HB (2012). Mitochondria, oligodendrocytes and inflammation in bipolar disorder: Evidence from transcriptome studies points to intriguing parallels with multiple sclerosis.. Neurobiol Dis.

